# Individual and Cumulative Effects of GWAS Susceptibility Loci in Lung Cancer: Associations after Sub-Phenotyping for COPD

**DOI:** 10.1371/journal.pone.0016476

**Published:** 2011-02-03

**Authors:** Robert P. Young, Raewyn J. Hopkins, Chris F. Whittington, Bryan A. Hay, Michael J. Epton, Gregory D. Gamble

**Affiliations:** 1 Department of Medicine, Auckland Hospital, Auckland, New Zealand; 2 Department of Medicine, University of Otago, Christchurch, New Zealand; 3 Synergenz Biosciences Ltd, Auckland, New Zealand; Ohio State University Medical Center, United States of America

## Abstract

Epidemiological studies show that approximately 20–30% of chronic smokers develop chronic obstructive pulmonary disease (COPD) while 10–15% develop lung cancer. COPD pre-exists lung cancer in 50–90% of cases and has a heritability of 40–77%, much greater than for lung cancer with heritability of 15–25%. These data suggest that smokers susceptible to COPD may also be susceptible to lung cancer. This study examines the association of several overlapping chromosomal loci, recently implicated by GWA studies in COPD, lung function and lung cancer, in (n = 1400) subjects sub-phenotyped for the presence of COPD and matched for smoking exposure. Using this approach we show; the 15q25 locus confers susceptibility to lung cancer and COPD, the 4q31 and 4q22 loci both confer a reduced risk to both COPD and lung cancer, the 6p21 locus confers susceptibility to lung cancer in smokers with pre-existing COPD, the 5p15 and 1q23 loci both confer susceptibility to lung cancer in those with no pre-existing COPD. We also show the 5q33 locus, previously associated with reduced FEV_1_, appears to confer susceptibility to both COPD and lung cancer. The 6p21 locus previously linked to reduced FEV_1_ is associated with COPD only. Larger studies will be needed to distinguish whether these COPD-related effects may reflect, in part, associations specific to different lung cancer histology. We demonstrate that when the “risk genotypes” derived from the univariate analysis are incorporated into an algorithm with clinical variables, independently associated with lung cancer in multivariate analysis, modest discrimination is possible on receiver operator curve analysis (AUC = 0.70). We suggest that genetic susceptibility to lung cancer includes genes conferring susceptibility to COPD and that sub-phenotyping with spirometry is critical to identifying genes underlying the development of lung cancer.

## Introduction

Lung cancer and chronic obstructive pulmonary disease (COPD) are both lung diseases that result from the combined effects of smoking exposure and genetic susceptibility [1], [2]. Epidemiological studies show that although tobacco smoke exposure accounts for nearly 90% of cases, only 10–15% of smokers develop lung cancer while 20%–30% develop COPD [3]–[5]. Genetic factors might explain these observations as heritability of lung cancer and reduced FEV_1_ (forced expiratory volume in one second that defines COPD) is estimated to be 15–25% and 40–77% respectively [6], [7]. The presence of COPD, a disease characterized by airflow limitation secondary to lung remodelling (emphysema and small airways fibrosis), confers a 4-6 fold increased risk of lung cancer compared to smokers (a) with normal lung function [8] or (b) randomly recruited from the community [9]. Studies also show that the distribution of FEV_1_ is bi-modal in heavy smokers and uni-modal in light smokers, supporting a genetic basis to COPD and the lung remodelling (FEV_1_) response to chronic smoking exposure [10]–[12]. Importantly, between 50–90% of those with lung cancer have pre-existing COPD, compared to 15% in randomly selected community-based smoking controls [8], [13]–[15]. This means lung cancer is not just a “complex disease” from a genetic perspective but that it is also a mixed phenotype that includes COPD as a sub-phenotype. The question that then arises is “Are the genetic effects underlying COPD also important in susceptibility to lung cancer?”

Recent genome-wide association (GWA) studies in lung cancer, COPD and lung function (FEV_1_) have reported significant associations at several chromosomal loci [16]–[23]. Interestingly, several of these loci (and implicated candidate genes) are common to both COPD and lung cancer, suggesting the possibility that shared pathogenetic pathways may underlie susceptibility to these diseases (Table 1). The above epidemiological and genetic findings suggest that lung cancer and COPD are not discrete diseases related only through smoking exposure, but that many of the smokers who are susceptible to COPD might also be susceptible to lung cancer [8], [12], [24]–[28]. Such a suggestion was made by Dr Tom Petty 5 years ago [24] and recently reviewed by Punturieri et al. [29]. Given the apparent overlap in susceptibility loci, it appears plausible that some of the genetic factors implicated in COPD might also be relevant in lung cancer [24]–[29]. This is analogous to the inter-related pathways underlying obesity and type 2 diabetes, where the FTO (Fat mass and obesity associated) gene has been implicated in both diseases [30]. In this context BMI is the physiological biomarker used to define the sub-phenotype of obesity just as FEV_1_ defines COPD. The question that then arises is “Given the possible overlap in genetic susceptibility between COPD and lung cancer, is there an alternative study design to current approaches that might better identify susceptibility genes in lung cancer?”

**Table 1:** Chromosomal loci associated with COPD, reduced lung function and Lung Cancer identified by GWA studies and overlap suggested by case-control study.

Disease	Chromosomal loci	Candidate genes	GWA Study Reference*
**COPD (FEV_1_)**	1q23	IL6R	Wilk et al. (16)
	4q22	FAM13A	Hancock et al. (23)Cho et al. (63)
	4q24	GSTCD	Repapi et al. (22)Hancock et al. (23)
	4q31	HHIP/GYPA	Wilk et al. (16)Pillai et al. (20)Repapi et al. (22)Hancock et al. (23)
	5q33	HTR4/ADAM19	Repapi et al. (22)Hancock et al. (23)
	6p21	BAT3/AGER	Repapi et al. (22)Hancock et al. (23)
	6q24	GPR126	Hancock et al. (23)
	15q25	CHRNA 3/5	Pillai et al. (20)
**Lung Cancer**	1q21	CRP	Amos et al. (17)
	4q31	GYPA	Amos et al. (17)
	5p15	CRR9 (TERT)	Amos et al. (17)Hung et al. (18)
	6p21	BAT3	Amos et al. (17)Hung et al. (18)
	6q24	RGS17§	You et al. (81)
	15q25	CHRNA 3/5	Amos et al. (17)Hung et al. (18)Thorgeirsson et al (19)
**COPD and Lung Cancer overlap**			**Case-control Reference**
	15q25	CHRNA 3/5	Young et al. (26)
	4q31	HHIP/GYPA	Young et al. (28)
	4q22	FAM13A	Young et al. (64)

*Available at www.genome.gov/gwastudies. Accessed 25/03/2010.

§ Associated with familial lung cancer [81].

The above observations suggest that an alternate genetic model to current case-control studies could be used for disease gene discovery in lung cancer [31]. This model would be different from that used in the recent GWA case-control studies [17]–[19], where genetic effects are explored in lung cancer cases and smoking controls with unknown, but likely different, COPD prevalence [26], [27], [32], [33]. With regards to the latter, the possibility that co-existing COPD in lung cancer cases might introduce an interactive or confounding effect in lung cancer association studies has been raised [26], [34]. To better understand the complex relationship between COPD and lung cancer, smokers in both cases and controls would ideally be matched for smoking exposure and sub-phenotyped for COPD using spirometry. Lung function testing is necessary to define this phenotype as COPD is insidious in onset and, due to a widespread underutilisation of spirometry, under-diagnosed in 50-80% of cases [9], [33]. Sub-phenotyping for COPD would then define three smoking cohorts, those with normal lung function (“resistant” controls), those with COPD and those with lung cancer sub-phenotyped for co-existing COPD. Using such an approach, the authors have shown that the chromosome 15q25 locus, originally associated with lung cancer in GWA studies [17]–[19], is also associated with COPD [26]. This observation has been subsequently replicated in both GWA [20] and candidate gene studies [35]. Using this same approach, the authors have also shown that the chromosome 4q31 locus, associated with a reduced risk of COPD [21]–[23], is also independently associated with a reduced risk of lung cancer [28].

The lung cancer, lung function and COPD GWA studies have identified to date at least nine chromosomal regions and eleven candidate genes (Table 1) that appear to be associated with COPD, lung function and/or lung cancer (1q23 [16], 4q22 [23], 4q24 [22], [23], 4q31 [17], [20]–[23], 5p15 [17], [18], 5q33 [22], [23], 6p21 [17]–[19], [22], [23] and 15q25 [17]–[21]). The question arises, “How do these loci affect susceptibility to lung cancer after sub-phenotyping for COPD and can they be combined to define a high risk smoker?” With this question in mind, we used the sub-phenotyping approach described above to examine the individual and cumulative effect of recently identified GWA loci implicated in both COPD (lung function) [20]–[23] and lung cancer [1], [17]–[19] studies. Using an algorithm from a previously published model, that includes age, family history of lung cancer and the prior diagnosis of COPD [27], [32], we combined both susceptible and protective genotypes from this analysis to derive and validate a risk score for susceptibility to lung cancer.

## Materials and Methods

### Study subjects

The subjects in this study have been previously described [26]. In brief, subjects were of Caucasian ancestry based on their grandparents' descent (all four grandparents of Caucasian descent). Lung cancer and COPD cases were recruited from a tertiary hospital clinic between 2000 and 2007 in Auckland while healthy smoking controls were recruited from the same community after volunteering for screening spirometry. Inclusion criteria were Caucasian ancestry (see above), aged 40 years or more and past smoking history (see below) while those unable to adequately perform spirometry were excluded (approximate 5% failure rate in each group). All participants gave written informed consent, and underwent blood sampling for DNA extraction, pre-bronchodilator spirometry and an investigator-administered questionnaire. Spirometry was performed using a portable spirometer (Easy-One™; ndd Medizintechnik AG, Zurich, Switzerland). Lung function conformed to American Thoracic Society (ATS) standards for reproducibility (http://www.thoracic.org/statements/), with the highest value of the best three acceptable blows used for classification of COPD status. COPD was defined according to Global Initiative for Chronic Obstructive Lung Diseases (GOLD) stage 2 or more criteria (FEV1/FVC<70% and FEV1% predicted ≤80%) using pre-bronchodilator spirometric measurements [www.goldcopd.com]. A modified ATS respiratory questionnaire (http://www.thoracic.org/statements/ was used which collected demographic data including age, sex, medical history, family history of lung disease, history of active and passive tobacco exposure, respiratory symptoms and occupational aero-pollutant exposures.

#### Lung cancer cohort

Subjects with lung cancer were recruited from a tertiary hospital clinic [26], aged >40 yrs and the diagnosis confirmed through histological or cytological specimens in 95% of cases. Non-smokers with lung cancer were excluded from the study and only primary lung cancer cases with the following pathological diagnosis were included: adenocarcinoma, squamous cell cancer, small cell cancer and non-small cell cancer (generally large cell or bronchoalveolar subtypes). Lung function measurement (pre-bronchodilator) was performed within 3 months of lung cancer diagnosis, prior to surgery and in the absence of pleural effusions or lung collapse on plain chest radiographs [8]. For lung cancer cases that had already undergone surgery, pre-operative lung function performed by the hospital lung function laboratory was sourced from medical records.

#### COPD cohort

Subjects with COPD were identified through hospital specialist clinics as previously described [26]. Subjects recruited into the study were aged 40–80 yrs, with a minimum smoking history of 20 pack-yrs and COPD confirmed by a respiratory specialist based on pre-bronchodilator spirometric criteria (GOLD stage 2 or more).

#### Control cohort

Control subjects were recruited based on the following criteria: aged 40–80 yrs and with a minimum smoking history of 20 pack-yrs. Control subjects were volunteers who were recruited from the same patient catchment area (residential area) as those serving the lung cancer and COPD hospital clinics through either (a) a community postal advertisement or (b) while attending community-based retired military/servicemen's clubs. Controls with COPD, based on pre-bronchodilator spirometry (GOLD stage 1 or more), who constituted 35% of the smoking volunteers, were excluded from further analysis.

The study was approved by the Multi Centre Ethics Committee (New Zealand).

### Study design

**Figure 1:**
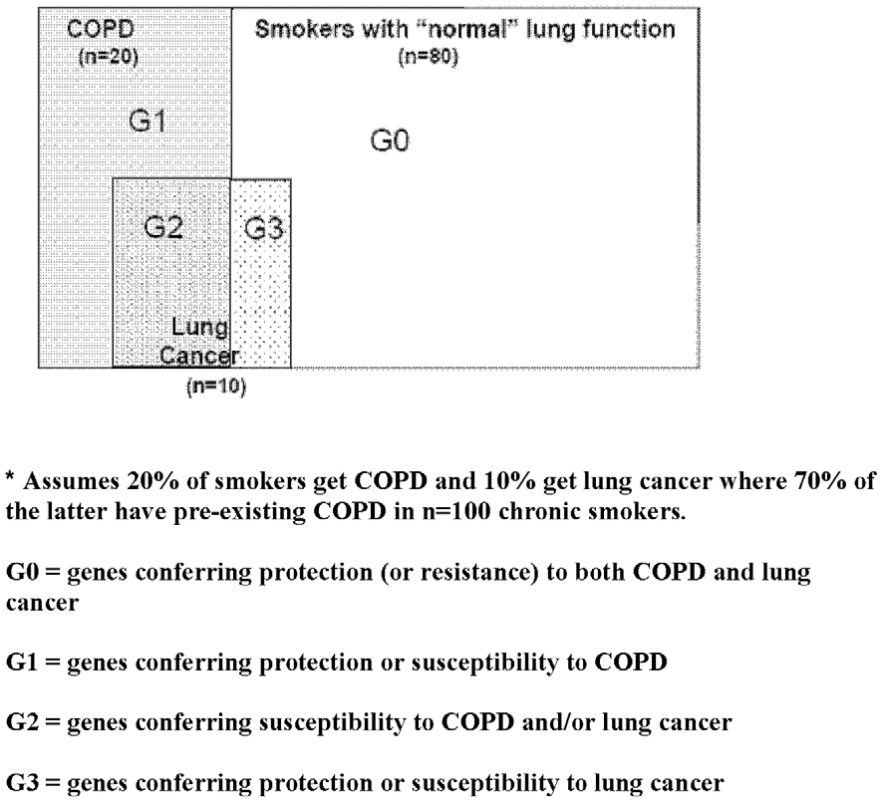
Genes conferring resistance (G0) and susceptibility to COPD (G1), lung cancer (G3) or both (G2): a pharmacogenetic approach to chronic smoke exposure*.

The present cross-sectional case–control study compared smokers of the same ethnicity with comparable demographic variables (specifically age, sex and smoking history). The controls in the current study were carefully chosen to best represent the majority of smokers who have maintained normal or near-normal lung function despite decades of smoking (“resistant smoker”) as shown by many studies [4], [5], [10]–[12]. Accordingly, the resistant smoker group best reflects those smokers least likely to develop lung cancer or COPD, thus minimising phenotype misclassification and improving the power to detect differences between affected and unaffected smokers [36]. We hypothesised that SNP associations might identify protective or susceptibility effects to one or a combination of COPD only (G1), COPD and lung cancer (G2), lung cancer only (G3) or neither disease (G0) (see Figure 1).

### Genotyping

Genomic DNA was extracted from whole blood samples using standard salt-based methods and purified genomic DNA was aliquoted (10 ng·µL^–1^ concentration) into 96-well or 384-well plates. Samples were genotyped using either the Sequenom™ system (Sequenom™ Autoflex Mass Spectrometer and Samsung 24 pin nanodispenser) by the Australian Genome Research Facility (www.agrf.com.au) or by our university lab using Taqman® SNP genotyping assays (Applied Biosystems, USA) utilising minor groove-binder probes. The Sequenom™ sequences were designed in house by AGRF with amplification and separation methods (iPLEX™, www.sequenom.com) as previously described [26], [27], [32]. Taqman® SNP genotyping assays were run in 384-well plates according to the manufacturer's instructions. PCR cycling was performed on both GeneAmp® PCR System 9700 and 7900HT Fast Real-Time PCR System (Applied Biosystems, USA) devices. SNP primers were designed by Applied Biosystems. Real-time amplification plots of selected plates were used to verify end-point allelic discrimination to establish reliability of the Taqman based genotyping.

The present study investigated the genotype frequencies of 11 SNPs. The rs16969968 SNP, situated within the nicotinic acetylcholine receptor (nAChR) gene on 15q25, the rs1052486 SNP, situated within the HLA-B associated transcript (BAT3) gene on 6p21, and the rs402710 SNP, situated within the cisplatin-resistance regulated gene 9 (CRR9) gene on 5p15, were genotyped using the Sequenom™ system, whilst the remaining eight SNPs, the rs7671167 SNP, situated within the Family with sequence similarity 13A (FAM13A) gene on 4q22, the rs1489759 SNP, situated near the hedgehog-interacting protein (HHIP) gene on 4q31, the rs2202507 SNP, situated near the glycophorin A (GYPA) gene on 4q31, the rs2808630 SNP, situated near the C-reactive protein (CRP) gene on 1q21, the rs10516526 SNP, situated within the glutathione S-transferase C-terminal domain (GSTCD) gene on 4q42, the rs1422795 SNP, situated within the A Disintegrin and Metalloproteinase 19 (ADAM19) gene on 5q33, the rs2070600 SNP, situated within the receptor for advanced glycation end-products (AGER) gene on 6p21, and the rs11155242 SNP, situated within the G-protein receptor 126 (GPR126) gene on 6q24, were genotyped by Taqman® SNP genotyping assays. Failed samples were repeated until call rates of ≥95% for each SNP in each cohort were achieved. Genotype frequencies for each SNP were compared between the 3 primary groups (control smokers, COPD and lung cancer cohorts) and with sub-phenotyping the lung cancer cohort according to the presence or absence of COPD based on GOLD 2 criteria.

### Algorithm and susceptibility score

The cumulative effect of those SNP genotypes identified as susceptible (Odds ratio, OR>1) or protective (OR<1), based on significant distortions in frequency (P<0.05) between the cases or sub-phenotypes and the control smokers, was examined using a previously published algorithm [27], [32]. Only the lung cancer and control smoker cohorts were used for this analysis. In this algorithm, for each subject, a numerical value of −1 was assigned for each of the protective genotypes present among the protective SNPs and +1 for each of the susceptible genotypes present. Where an individual did not have either the protective or susceptibility genotype for that SNP, the score was 0 (i.e. did not contribute to the genetic score). This approach is consistent with a recently published study in prostate cancer [37]. As previously described [27], [32], weighting the presence of specific susceptible or protective genotypes according to their individual odds ratios (ORs; from univariate regression) did not significantly improve the discriminatory performance of the cumulative SNP score (unpublished data).

The algorithmic approach used here involved deriving an overall “susceptibility score” for each subject (from the control and lung cancer cohorts) by combining genetic data (cumulative SNP scores) and clinical variables, identified in a multivariate analysis as previously described [27], [32]. The clinical variables (and score) were age >60 years of age (+4), family history of lung cancer (+3) and prior diagnosis of COPD (+4) [32]. By using multivariate logistic and stepwise regression analysis, the 9-SNP panel was examined in combination with the pre-stipulated clinical variables above. As smoking exposure (pack-yrs) was a recruitment criterion for this study, and comparable between cases and controls, it was not included in the scoring system described here. The lung cancer susceptibility score (for the control and lung cancer cohorts) was plotted with (*a*) the frequency of lung cancer and (*b*) the floating absolute risk (FAR, equivalent to OR) across the combined smoker/ex-smoker cohort [38], [39]. The FAR approach was adopted since it uses a ‘floated’ variance across all polychotomous risk categories rather than choosing on referent level and enables confidence intervals to be presented for all risk categories.

### Analysis

Patient characteristics in the cases and controls were compared by ANOVA for continuous variables and Chi-squared test for discrete variables (Mantel–Haenszel, odds ratio (OR)). Genotype and allele frequencies were checked for each SNP by Hardy–Weinberg Equilibrium (HWE). Population admixture across cohorts was performed using structure analysis on genotyping data from 40 unrelated SNPs [40]. Distortions in the genotype and allele frequencies were identified by comparing lung cancer (sub-phenotyped by COPD) and/or COPD cases with “resistant” smoking controls using two-by-two contingency tables. Both the additive (allelic) and genotype based genetic models were tested although the latter is preferred [41]. Correction for multiple comparisons was not done as the SNPs were selected “a priori” from the GWA studies. Individual SNPs were not included in the combined risk model on the basis of statistical significance shown here but were included because they were identified by the GWA studies to be highly significantly associated with lung cancer. In this respect, this study was sufficiently powered to enable a small level of discrimination between cases and controls to be demonstrated for the resultant overall model rather than individual SNPs. With at least 450 cases and 450 controls this study achieves 80% likelihood to detect an area under the ROC curve of 0.55 using a two-sided z-test at the 5% significance level, ie we can conclude that the ROC curve for the SNP model offers better than chance association when the area under the receiver operating characteristics curve is at least 0.55 (Hintze, J (2006) PASS 2002, WWW.NCSS.COM)

Genotype data (9-SNP panel) and the clinical variables were combined in a stepwise logistic regression to assess their relative effects on discriminating low and high risk (by point estimate and receiver operating characteristic (ROC) curve) by score quintile. The frequency distribution of the lung cancer susceptibility score was compared across the cases and controls. Its clinical utility was assessed using ROC analysis, which assesses how well the model predicts risk across the score (i.e. clinical performance of the score with respect to sensitivity, and specificity).

## Results

### Demographic variables

Characteristics of the lung cancer cases, COPD cases and healthy control smokers are summarized in Table 2. The demographic variables and histological subtypes of the lung cancer cases are comparable to previously published data [42]. The staging at diagnosis was also comparable to this published series (data not shown) suggesting the lung cancer cohort is representative. The COPD cases have higher pack-year exposure than the lung cancer cases and healthy control smokers (P<0.05). This reflects outliers with high smoking histories in the COPD cohort that after log transformation of pack-years showed all groups were comparable (data not shown). All groups are comparable with respect to age started smoking, years smoked, years since quitting and cigarettes/day (Table 2). Overall, we believe the three groups are well matched for smoking exposure. We note a lower frequency of current smokers in the lung cancer and COPD cohorts, compared to healthy smokers (35% vs 40% vs 48% respectively) which may reflect an effect from their smoking-related diagnosis. Current smoking status had no effect on the lung function in the lung cancer cases group. The lung cancer cases, COPD cases and smoking controls were also comparable with respect to other aero-pollutant exposures (Table 2). Those with lung cancer had a higher prevalence for a positive family history of lung cancer compared to the COPD cases and healthy smokers (19% vs 11% vs 9%). As expected, lung function was worse in the lung cancer and COPD cohorts compared to the healthy smoker controls. Testing lung function in the lung cancer cases (as described above) enabled stratification of results to test for an interactive or confounding effect of COPD.

**Table 2:** Summary of characteristics for the lung cancer and resistant smokers.

Parameter Mean (1 SD)	Lung Cancer N = 454	COPD N = 458	Control smokers N = 488
% male	53%	59%	60%
Age (yrs)	69 (10)	66 (9)	65 (10)
Height (m)*	1.67 (0.08)	1.68 (0.09)	1.69 (0.09)
**Smoking history**			
Current smoking (%)	35%	40%	48%
Age started (yr)	18 (4)	17 (3)	17 (3)
Yrs smoked	41 (12)	42 (11)	35 (11)
Pack-years*	41 (25)	47(20) §	40 (19)
Cigarettes/day	20 (10)	23 (9)	24 (11)
**Yrs since quitting**	11.4 (6.7)	9.8 (7.4)	13.9 (8.1)
**History of other exposures**			
Work dust exposure*	63%	59%	47%
Work fume exposure	41%	40%	38%
Asbestos exposure*	23%	22%	16%
**Family history**			
FHx of COPD	33%	37%	28%
FHx of lung cancer*	19%	11%	9%
**Lung function**			
FEV1 (L)*	1.86 (0.48)	1.25 (0.48)	2.86 (0.68)
FEV1 % predicted*	73%	46%	99%
FEV1/FVC*	64% (13)	46% (8)	78% (7)
Spirometric COPD# *	51%	100%	0%

#According to GOLD 2+ criteria,

*P<0.05.

§ No significant difference after log transformation of pack- years due to skewed distribution.

### Genotyping

The genotyping results for the 12 SNPs are shown in Table 3. The allele and genotype frequencies were comparable to those reported in the literature and from the International Hapmap Project (www.hapmap.org). The observed genotypes for the two Chr 4q31 SNPs (HHIP and GYPA) in this study were 65% concordant, in accordance with the reported degree of LD between these SNPs. The concordance for the other SNPs in “close” proximity (BAT3 and AGER on 6p21) showed very poor concordance as expected. As all SNPs were in Hardy-Weinberg equilibrium and amplification plots were used to ensure correct genotype calls, significant genotyping error is unlikely. We found no evidence for population stratification between the cohorts using 40 unlinked SNPs from unrelated genes (mean χ^2^ = 3.3, P = 0.58) [40]. Based on distortions in genotype frequency between the 3 groups, risk genotypes were assigned as generally conferring protection or susceptibility to COPD and/or lung cancer according to Figure 1.

**Table 3:** Genotype frequencies for the candidate SNP identified by GWA studies of COPD, lung function and lung cancer.

Chromosomeloci	Candidate SNP(rs)	Genotypes	Primary Cohorts			Lung cancer (LC)Sub-phenotyped for COPD#	
			“Resistant”	“Susceptible”			
			ControlsN = 484	COPDN = 455	Lung CancerN = 446	LC + COPDN = 215	LC onlyN = 207
1q23	CRP(rs 2808630)	TTTCCC	225 (47%)205 (42%)53 (11%)	214 (48%)197 (44%)35 (8%)	214 (49%)193 (44%)34 (8%)	99 (48%)90 (43%)18 (9%)	106 (52%)85 (42%)11 (5%)*
		CC vs TT/TC	OR (95% CI)P value	0.69 (0.43–1.11)P = 0.10	0.68 (0.42–1.09)P = 0.09	0.77 (0.42–1.40)P = 0.37	0.47 (0.22–0.95)P = 0.02 §
4q22	FAM13A(rs 7671167)	CCTCTT	145 (30%)240 (49%)100 (21%)	107 (23%)*234 (51%)117 (26%)	96 (21%)*235 (52%)118 (26%)	47 (22%)*118 (55%)50 (23%)	41 (20%)*103 (50%)63 (30%)
		CC vs TT/TC	OR (95% CI)P value	0.71 (0.53–0.97)P = 0.02 §	0.64 (0.47–0.87)P = 0.003 §	0.66 (0.44–0.97)P = 0.03 §	0.58 (0.38–0.87)P = 0.006 §
4q24	GSTCD(rs 10516526)	AAAGGG	409 (85%)69 (14%)1 (0.2%)	394 (86%)61 (13%)2 (0.4%)	381 (86%)63 (14%)0 (0%)	178 (83%)37 (17%)0 (0%)	180 (89%)23 (11%)0 (0%)
		GG/AG vs AA	OR (95% CI)P value	0.93 (0.64–1.37)P = 0.72	0.97 (0.66–1.42)P = 0.85	1.21 (0.77–1.92)P = 0.38	0.75 (0.44–1.27)P = 0.25
4q31	HHIP(rs 1489759)	AAAGGG	178 (37%)223 (46%)83 (17%)	187 (41%)220 (48%)50 (11%)*	174 (39%)215 (48%)56 (13%)*	103 (48%)86 (40%)24 (11%)*	97 (47%)82 (40%)27 (13%)
		GG vs AA/AG	OR (95% CI)P value	0.59 (0.40–0.88)P = 0.006 §	0.70 (0.47–1.02)P = 0.05	0.61 (0.39–1.02)P = 0.05 §	0.73 (0.44–1.19)P = 0.18 §
4q31	GYPA(rs 2202507)	AAACCC	138 (29%)213 (44%)129 (27%)	136 (30%)233 (51%)88 (19%)	116 (26%)233 (53%)90 (21%)*	62 (29%)107 (50%)43 (20%)	52 (25%)113 (55%)39 (19%)*
		CC vs AA/AC	OR (95% CI)P value	0.65 (0.47–0.89)P = 0.06	0.70 (0.51–0.97)P = 0.02	0.69 (0.46–1.04)P = 0.06	0.64 (0.42–0.98)P = 0.03
5p15	CRR9 (TERT) (rs 402710)	GGGAAA	216 (44%)230 (47%)41 (8%)	200 (44%)206 (45%)52 (11%)	212 (47%)198 (44%)43 (9%)	90 (42%)106 (49%)19 (8%)	109 (53%)*77 (37%)21 (10%)
		GG vs GA/AA	OR (95% CI)P value	0.97 (0.75–1.27)P = 0.83	1.10 (0.85–1.44)P = 0.45	0.90 (0.64–1.27)P = 0.54	1.4 (0.99–1.96)P = 0.05
5q33	HTR4(rs 11168048)	TTCTCC	160 (33%)228 (47%)98 (20%)	153 (33%)216 (47%)89 (19%)	155 (34%)209 (46%)88 (19%)	80 (37%)95 (44%)40 (19%)	61 (29%)101 (49%)45 (22%)
		CC vs TT/TC	OR (95% CI)P value	0.95 (0.68–1.33)P = 0.78	0.96 (0.69–1.34)P = 0.79	0.90 (0.59–1.39)P = 0.63	1.10 (0.72–1.67)P = 0.64
5q33	ADAM19(rs 1422795)	TTCTCC	213 (44%)227 (47%)46 (9%)	189 (42%)207 (47%)59 (13%)	183 (41%)210 (47%)58 (13%)	86 (40%)100 (47%)29 (13%)	84 (41%)96 (47%)26 (13%)
		CC vs TT/TC	OR (95% CI)P value	1.47 (0.96–2.26)P = 0.07	1.44 (0.94–2.23)P = 0.08	1.51 (0.89–2.55)P = 0.10	1.40 (0.81–2.41)P = 0.20
6p21	BAT3(rs 1052486)	AAAGGG	119 (26%)239 (51%)108 (23%)	127 (29%)222 (50%)93 (21%)	112 (26%)210 (48%)116 (26%)	51 (24%)93 (44%)65 (31%)*	55 (27%)105 (52%)43 (21%)
		GG vs AA/AG	OR (95% CI)P value	0.88 (0.64–1.22)P = 0.44	1.19 (0.87–1.63)P = 0.25	1.50 (1.02–2.19)P = 0.03	0.89 (0.59–1.35)P = 0.57
6q21	AGER(rs 2070600)	CCCTTT	412 (85%)70 (14%)3 (0.6%)	413 (90%)41 (9%)*3 (0.7%)*	388 (87%)58 (13%)2 (0.4%)	185 (86%)29 (13%)1 (0.5%)	175 (86%)28 (14%)1 (0.5%)
		TT/TC vs CC	OR (95% CI)P value	0.60 (0.40–0.91)P = 0.01 §	0.87 (0.59–1.28)P = 0.47	0.92 (0.56–148)P = 0.71	0.94 (0.57–1..52)P = 0.78
6q24	GPR126(rs 11155242)	AAACCC	298 (63%)161 (34%)14 (3%)	290 (65%)147 (33%14 (3%)	287 (64%)147 (33%)11 (3%)	141 (66%)69 (32%)3 (1%)	128 (62%)69 (34%)8 (4%)
			OR (95% CI)P value	1.05 (0.47–2.36)P = 0.90	0.83 (0.35–1.97)P = 0.65	0.47 (0.11–1.76)P = 0.23	1.33 (0.50–3.45)P = 0.53
15q25	CHRNA 3/5 α(rs 16969968)	GGGAAA	225 (47%)205 (43%)45 (9%)	166 (37%)219 (49%)60 (14%)	170 (39%)199 (46%)68 (16%)*	86 (33%)125 (48%)50 (19%)*	81 (48%)69 (41%)18 (11%)
		AA vs GG/GA	OR (95% CI)P value	1.47 (0.97–2.29)P = 0.06 §	1.76 (1.16–2.68)P = 0.005 §	2.26 (1.43–3.58)P = 0.002 §	1.15 (0.62–2.11)P = 0.64 §

# COPD defined according to pre-bronchodilator GOLD 2+ spirometry criteria.

*P-value of genotype/s - cases vs controls <0.05.

§ P-value of risk allele - cases vs controls <0.05. Risk alleles are: CRP-C, FAM13A-C, HHIP-G, AGER-C, CHRNA3/5 α-A.

### Genotype associations according to sub-phenotyping for COPD (Table 3)

The results below describe individual SNP associations between resistant smokers and those with COPD or lung cancer (total and subdivided by co-existing COPD). We found no effects from gender, height or smoking status (current vs former) on any of these associations. A relationship between SNP variants and lung function was only found for rs 16969968 in the lung cancer cases as previously published (26) but not for the other SNP variants (unpublished data). The numbers were considered too small to look at lung cancer sub-grouped by histology. The genotype results below are summarised in Table 3.

#### Rs16969968, 15q25 (CHRNA 3/5)

As previously reported [26], compared to controls the AA genotype was more frequently found in lung cancer cases (N = 454, 16% vs 9%, OR = 1.76, P = 0.005) COPD cases (N = 458, 14% vs 9%, OR = 1.47, P = 0.06) and for all COPD cases (GOLD 2+) with or without lung cancer (N = 706, 16% vs 9%, OR = 1.76, P = 0.002). More importantly, when the lung cancer cases were sub-phenotyped into those with and without COPD (GOLD 2+ criteria, n = 429), the frequency of the AA genotype was quite different: 19% (vs 9% in controls, OR = 2.26, P = 0.002) and 11% (vs 9% in controls, OR = 1.15, P = 0.64) respectively (Table 3). Based on the data to date, the AA genotype of the CHRNA 3/5 SNP most likely confers susceptibility to both lung cancer and COPD (G2 in Figure 1 and Table 4).

**Table 4:** Summary of the frequencies of the “risk genotype” for the 9 SNP panel for lung cancer susceptibility.

Candidate Gene	ChromosomeLocus	RiskGenotype	Controls	COPD	Lung Cancer	LC + COPD	LC only	Genotype effect
**CHRNA 3/5**(rs16969968)	**15q25**	**AA**(susceptible)	9%	14%	↑16%*	↑19%*	11%§	G2
**BAT3**(rs1052486)	**6p21**	**GG**(susceptible)	23%	21%	26%	↑31%*	21%§	G2
**CRR9 (TERT)**(rs402710)	**5p15**	**GG**(susceptible)	44%	44%	47%	42%	↑53%* §	G3
**HHIP**(rs1489759)	**4q31**	**GG**(protective)	17%	↓11%*	↓13%*	↓11%*	13%	G0
**GYPA**(rs2202507)	**4q31**	**CC**(protective)	27%	↓19%*	↓21%*	↓20%*	↓19%*	G0
**FAM13A**(rs 7671167)	**4q22**	**CC**(protective)	30%	↓23%*	↓21%*	↓22%*	↓20%*	G0
**ADAM 19**(rs1422795)	**5q33**	**CC**(susceptible)	9%	↑13%	↑13%	13%	13%	G2
**AGER**(rs2070600)	**6p21**	**CT/TT**(protective)	15%	↓10%*	13%	14%	15%	G1
**CRP**(rs2808630)	**1q23**	**CC**(protective)	11%	8%	8%	9%	↓5%*	G3

*P-value <0.05 for the risk genotype vs non-risk genotype/s compared to matched smoking controls (Mantel-Haenszel).

§ P value <0.05 for the risk genotype vs non-risk genotype/s comparing LC only to LC+ COPD (Mantel-Haenszel).

**↑ increased in cases compared to controls, ↓ in cases compared to controls.**

**G0: protective against COPD and lung cancer, G1: associated with COPD only, G2: associated with both lung cancer and COPD, G3: associated with lung cancer only.**

#### Rs7671167, 4q22 (FAM13A)

Consistent with previous studies, the CC genotype was found more frequently in control subjects compared to those with COPD (N = 458, 30% vs. 23%, OR = 0.71, P = 0.024) (63), lung cancer (N = 454, OR = 0.64, P = 0.003) (Table 3) lung cancer with COPD cases excluded (N = 207, OR = 0.58, P = 0.006) and lung cancer with COPD (N = 215, OR = 0.66, P = 0.03). No association was found with lung function among the lung cancer cases. The CC genotype of the FAM13A SNP appears to confer protection against both COPD and lung cancer (G0 in Figure 1 and Table 4).

#### Rs1052486, 6p21 (BAT3)

The GG genotype was 23% in the controls group compared to 26% in the lung cancer group (N = 454, OR = 1.19, P = 0.25) and 21% in the COPD group (N = 458, OR = 0.88, P = 0.44) (Table 4). Compared to controls, the GG genotype was significantly greater in those with lung cancer and COPD (N = 215) (23% vs 31%, OR = 1.50, P = 0.03) but no different in the lung cancer only subgroup (N = 207) (23% vs 21%, OR = 0.89, P = 0.57). The GG genotype was significantly greater in the lung cancer with COPD group than the lung cancer only group (31% vs 21%, OR = 1.68, P = 0.02). The GG genotype of the BAT3 SNP appears to confer susceptibility for lung cancer in those with COPD (G2 in Table 4).

#### Rs402710, 5p15 (CRR9/TERT)

We found no difference in the GG genotype frequency in controls and COPD cases (44% vs 44%, OR = 0.97, P = 0.83) or lung cancer cases (44% vs 47%, OR = 1.10, P = 0.45) (Table 4). Compared to controls, the GG genotype was significantly higher in lung cancer cases only (N = 207, 44% vs 53%, OR = 1.40, P = 0.05) but not in lung cancer cases with COPD (44% vs 42%, OR = 0.90, P = 0.54) (Table 4). The GG genotype is significantly greater in the lung cancer only patients compared to the lung cancer with COPD group (53% vs 42%, OR = 1.54, P = 0.03). The GG genotype of the CRR9 (TERT) SNP appears to confer susceptibility for lung cancer only (G3 in Figure 1 and Table 4).

#### Rs1489759 and rs2202507, 4q31 (HHIP and GYPA respectively)

The GG genotype of the HHIP (rs 1489759) SNP was found to be more prevalent in the control group compared to COPD (17% vs 11%, OR = 0.59, P = 0.006) and lung cancer (17% vs 13%, OR = 0.70, P = 0.05) groups (Table 4). Similarly, the corresponding (minor) CC genotype of the GYPA (rs 2202507) SNP was more prevalent in the resistant smokers group compared to those with COPD (27% vs 19%, OR = 0.65, P = 0.06) and lung cancer (27% vs 21%, OR = 0.70, P = 0.02) groups (Table 4). When the lung cancer cases were stratified by available spirometric data (n = 419 and n = 416 for HHIP and GYPA genotyping, respectively), into those with and without COPD (GOLD 2+ criteria), the distribution of the minor allele homozygote for both SNPs does not change significantly. The effect sizes of the homozygote minor allele in these sub-analyses remain the same, although the p values are degraded due to smaller sample sizes. When grouping all subjects with COPD (combining COPD and lung cancer with COPD groups, N = 670), the protective effect was nearly identical to that from using the COPD cohort alone (OR = 0.60, P = 0.003 and OR 0.66, P = 0.004 for the HHIP and GYPA, respectively). The minor allele homozygotes for HHIP and GYPA SNPs (GG and CC, respectively) appear to confer protection from both lung cancer and COPD (G0 in Figure 1 and Table 4).

#### Rs1422795, 5q33, (ADAM19)

Compared to controls, the frequency of the CC genotype was marginally increased lung cancer cases (9% vs 13%, OR = 1.44, P = 0.08) and COPD cases (9% vs 13%, OR 1.47, P = 0.07) groups (Table 3). When the lung cancer cases were divided according to COPD the effect size remained the same although p-values were degraded due to smaller numbers (lung cancer with COPD 13%, OR = 1.51, P = 0.10 and lung cancer without COPD 13%, OR = 1.40, P = 0.20). When the CC genotype frequency of the controls is compared to those with COPD and lung cancer with COPD (9% vs 13%, OR = 1.45, P = 0.05) the larger cohort identifies a significant increase in the CC genotype in those with the COPD phenotype. The CC genotype is likely to be associated with modest susceptibility to both COPD and lung cancer (G2 in Figure 1 and Table 4).

#### Rs2070600, 6q21 (AGER)

Compared to controls, the TT/TC genotype frequency was significantly decreased in COPD patients (10% vs 15%, OR = 0.60, P = 0.01) but not in lung cancer (13% vs 15% in controls, OR = 0.87, P = 0.87). Sub-grouping lung cancer cases according to COPD phenotype did not identify any other associations. The TT/TC genotypes of the AGER SNP appeared to confer a protective effect for COPD (G1 in Figure 1 and Table 4).

#### Rs2808630, 1q23 (CRP)

ompared to controls, the CC genotype was slightly less frequent in lung cancer (11% in 8%, OR = 0.68, P = 0.09) and COPD groups (11% vs 8%, OR = 0.69, P = 0.10) but significantly lower in the lung cancer only group (11% in controls vs 5%, OR = 0.47, P = 0.02). The frequency of the CC genotype was also significantly lower in the lung cancer only cohort compared to lung cancer with COPD despite the modest numbers (5% vs 9%, OR = 0.54, P = 0.03). This suggests the CC genotype of the CRP SNP was associated with susceptibility to lung cancer only (G0 in Figure 1 and Table 4).

### Gene-based risk model

**Figure 2:**
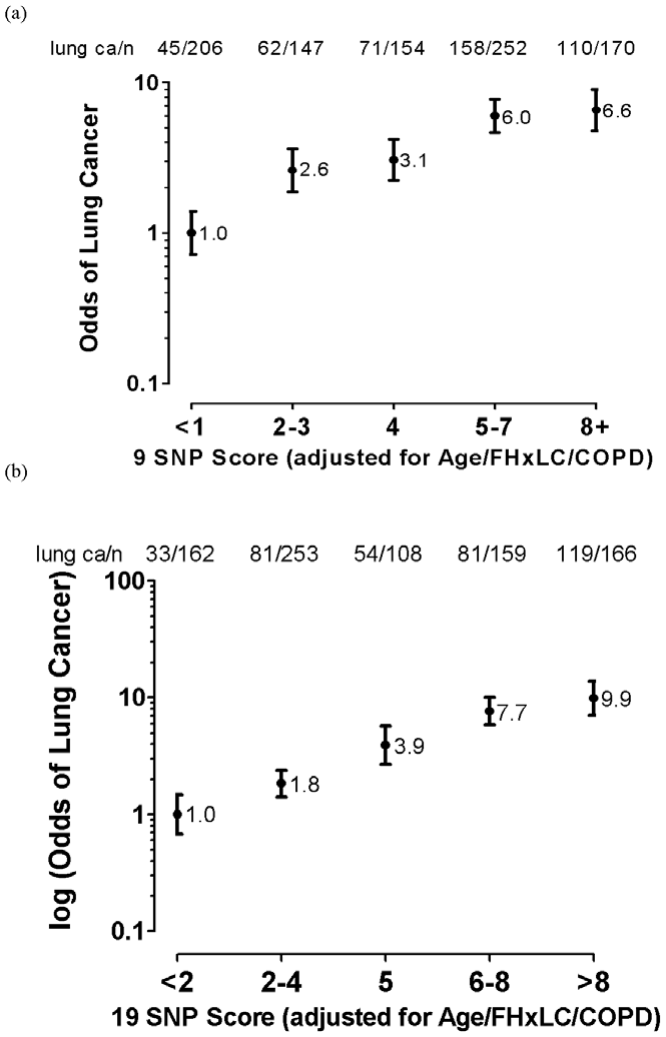
Cumulative effect of the (a) 9 SNP panel and (b) 19 SNP panel of protective and susceptible SNPs in combination with non-genetic variables to derive a “lung cancer risk score” in lung cancer cases and controls (n = controls and lung cancer cases combined).

**Figure 3:**
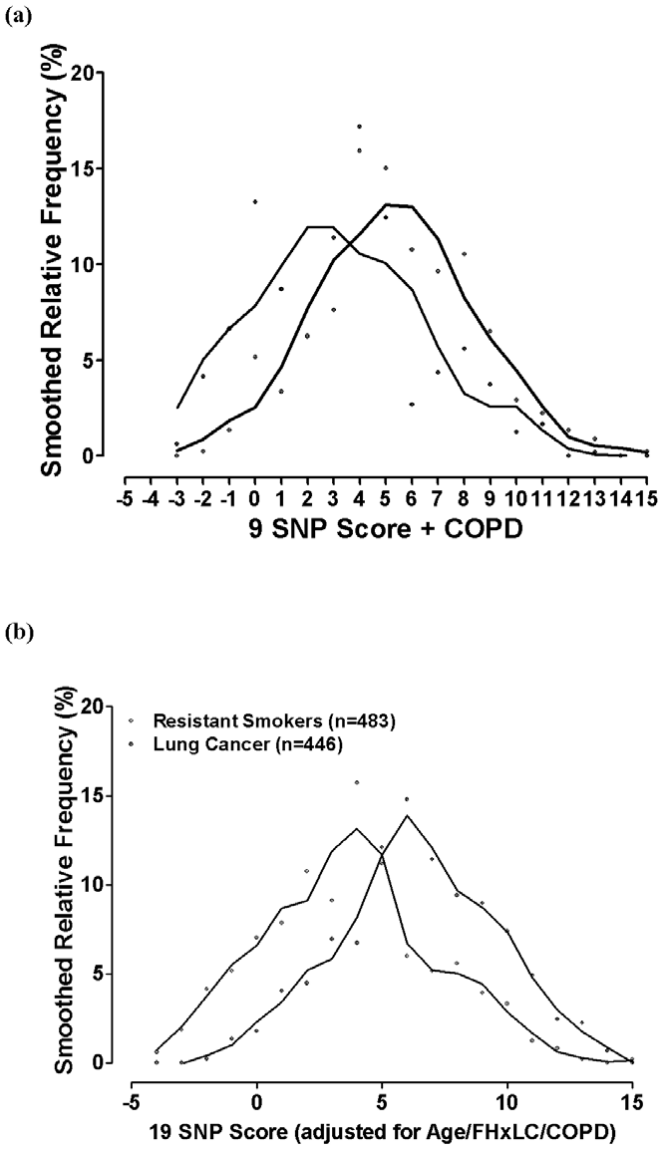
Distribution of the lung cancer susceptibility score using the (a) 9 SNP panel and (b) 19 SNP panel, of protective and susceptible SNPs in combination with non-genetic variables in lung cancer cases and controls.

Using the results of the uni-variate analysis above, nine “risk genotypes” were identified as either protective or susceptible (Table 4). For each subject in the smoking control and lung cancer cohorts, the sum total of these SNP-based scores were added to the scores for the clinical variables (age, diagnosis of COPD, family history of lung cancer) to derive a total lung cancer susceptibility score [27], [32]. On FAR analysis [25], [26], the plot of the total score with the frequency of lung cancer shows a linear relationship across SNP score quintiles for both the 9 SNP (Figure 2a) and 19 SNP (Figure 2b) panels, as previously shown [27], [32]. The distribution plot of the total scores according to control smokers (blue line, Figure 3) and lung cancer cases (red line, Figure 3) is bimodal and the corresponding AUC is 0.69 for the 9 SNP panel used here (Figure 3a). When genotype data of the 10 most significant SNPs (smallest P values) from a previous analysis [32] are added to the 9 SNP panel, the AUC increases to 0.72 (Figure 3b). We note when the clinical variables only are used the AUC is 0.67 compared to the 9 SNPs alone of 0.59 and 19 SNPs alone of 0.67. We conclude that the addition of the 9 SNPs or 19 SNPs improves the AUC and the risk prediction utility of the risk score.

## Discussion

This study provides further evidence that the genes underlying susceptibility to lung cancer may include genes relevant to susceptibility to COPD. This has been possible by using cohorts of smokers, matched for smoking exposure, but quite different in their phenotypic response to smoking exposure. This phenotypic response has been defined in part by the presence or absence of COPD, itself a common sub-phenotype of lung cancer [8], [13], [14], defined by a measurable biomarker (FEV_1_) with a strong genetic basis [2], [7]. By comparing chronic smokers with normal lung function with those with COPD and lung cancer, sub-phenotyped for COPD, the genetic associations identified to date can be better understood. Indeed, by re-examining the associations reported from recently reported lung cancer and COPD (FEV_1_) GWA studies, the results of this current study suggest the genetic effects from these loci confer specific protective or susceptibility effects on COPD, Lung cancer or both (Figure 1, Tables 1 and 4). Despite comparatively small sample sizes here, using this approach the authors have recently shown that the 15q25 (CHRNA 3/5) and 4q31 (HHIP/GYPA) loci might be relevant in both COPD and lung cancer [26], [28]. The results in this study suggest that the rs1052486 SNP on the 6p21 locus (BAT3) confers susceptibility to lung cancer in smokers with pre-existing COPD and that, the rs402710 SNP on the 5p15 locus (CRR9/TERT) and the rs2808630 SNP on the 1q23 locus (CRP) confer susceptibility to lung cancer in those with no pre-existing COPD. The rs1422795 SNP on the 5q33 locus (ADAM 19), previously associated with reduced FEV_1_ [22], [23], might also confer susceptibility to both COPD and lung cancer. The rs7671167 SNP on the 4q22 locus (FAM13A), previously linked to reduced lung function and COPD [23,] is associated with both COPD and lung cancer. Larger studies will be needed to confirm these findings as the sample sizes here are small, particularly after sub-phenotyping the lung cancer cases for COPD. These results also suggest that the previously published risk algorithm [27], [32], where combining risk genotypes and clinical variables identified in a multivariate analysis, can segment smokers into moderate, high and very high risk of lung cancer. The authors conclude that when spirometry is used to sub-phenotype smokers, genes with effects on reduced lung function or COPD appear to be relevant in “susceptibility” to lung cancer. This provides further evidence to support existing epidemiological studies suggesting COPD and lung cancer are related by more than smoking exposure [24], [30] but also an overlapping genetic susceptibility to smoking (Figure 1 and Tables 1 and 4) [26], [28].

Epidemiological studies suggest COPD is an important sub-phenotype of lung cancer. The results of this study suggest genetic associations broadly define three disease groups: smokers primarily susceptible to COPD (G1), smokers susceptible to both COPD and lung cancer (G2), and smokers susceptible to lung cancer only (G3) (Figure 1 and Table 4). More importantly, the epidemiological studies also show there is a fourth group of smokers, consisting of the majority of smokers (≈70%) [4], [5], [12], who maintain normal or near normal lung function. This group, have a “resistant” phenotype (G0), either do not develop, or are at least risk of, COPD and lung cancer [4], [5], [8], [9], [12]. This is likely to be due, in part, to an excess of protective genetic variants compared to susceptibility variants [27], [31]. Based on the results of this study, the G0 genes conferring protection from COPD and lung cancer include the rs7671167 SNP (FAM13A gene on the Chr 4q22 locus) and the rs1489759 and rs2202507 SNPs (GYPA and HHIP genes on the Chr 4q31 locus). The rs2070600 SNP (AGER on the Chr 6p21 locus), previously linked to reduced FEV_1_, appears to be a susceptibility gene for COPD but not lung cancer (G1). Both the rs169968 SNP (CHRNA3/5 gene on the Chr 15q25 locus) and the rs1052486 SNP (BAT3 gene on the Chr 6p21 locus) appear to confer susceptibility to lung cancer, but the latter only in conjunction with COPD (G2). The rs402710 SNP (CRR9 (TERT) on the Chr 5p15 locus) appears to confer susceptibility to lung cancer in those with no pre-existing COPD, in keeping with other studies (G3) [34], [43], [44]. These observations require validation in larger studies where SNP effects on histological subtypes might also be relevant to our findings [1], [43]. Several loci linked to lung function in the general population, such as the rs10516526, rs11168048 and rs11155242 SNPs (GSTCD on 4q24, HTR4 on 5q33, and GPR126 on 6q24, respectively) [22], [23] do not appear to be related to COPD in this study. However, given that the population study did not look specifically at smokers, it is possible that these loci are not relevant to the lung's response to tobacco smoke exposure. The authors conclude that the novel study design used here provides a viable approach with which to better understand the genetic epidemiology of lung cancer.

The pathologic link between COPD and lung cancer may stem in part from the overlapping inflammatory, apoptotic and matrix remodelling/repair processes [45]–[47] underlying COPD, and the development of squamous metaplasia, epithelial-mesenchymal transition (EMT) and DNA damage that underlies lung carcinogenesis [28], [45], [48]–[51]. In particular, there is growing evidence that suggests these smoking induced changes are orchestrated by the bronchial epithelium [28], [45], [48]–[51] - the HHIP, CHRNA 3/5 and FAM13A proteins are all known to be expressed on the bronchial epithelium (see below). Although several of the SNPs, investigated in this study have been shown to have functional effects on gene expression or protein function, they may not themselves be the causal variant, but instead representative of the causal allele through linkage disequilibrium [52]. We note that in many instances, the physical distance between these risk SNPs and the proposed candidate genes is large. Despite this, it remains possible that the investigated SNPs are themselves functional as (a) studies have shown that SNPs with regulatory effects on genes maybe some distance away [50], and (b) it has recently been recognised that common SNPs with consistent disease association signals, through “Synthetic associations”, may represent the biological effects of rare variants in nearby genes as much as 2 mega-bases apart [53]. If such an effect were true, then there is potential for considerable overlap between the susceptibility genes for COPD and for lung cancer. The rs16969968 SNP (CHRNA 3/5 on 15q25,) investigated in this study results in a non-synonymous amino-acid change in a highly conserved region of the second intra-cellular loop of the α5 subunit of the nicotinic acetylcholine receptor. This receptor is expressed on both bronchial epithelial cells and inflammatory cells, and is believed to moderate pulmonary inflammation [54] and lung destruction [34]. This receptor also binds both nitrosamines (known carcinogens in cigarette smoke [55]) and nicotine linking it to lung cancer and nicotine addiction respectively [56]. The rs1052486 SNP (BAT3 on 6p21,) is a missense mutation (Ser619Pro) in the BAT3 gene and has been previously linked to lung cancer [57]. BAT3 is a nuclear protein that influences apoptosis through it's interaction with p53 [58] linking it to both COPD and lung cancer. The rs1489759 SNP (HHIP on 4q31,) is 93 kb upstream of the HHIP gene and of unknown function. The HHIP protein is believed to be important in the bronchial epithelial response to smoking [59] and epithelial repair processes in lung cancer [60]. The HHIP protein has been linked with epithelial-mesenchymal transition, a pathological process that results from lung remodelling (with release of metalloproteinases and growth factors [29], [45], [61]) and initiates lung carcinogenesis [48]. The rs2202507 SNP (GYPA on 4q31,) is of unknown function and downstream of the GYPA gene. The GYPA protein, found on erythrocytes, shows reduced expression in COPD and is indicative of oxidative stress [62]. Whether the GYPA association with COPD and lung cancer reflects an independent effect or linkage effect with the HHIP locus (LD≈0.70) is still debated [21]. The rs7671167 SNP (FAM13A on 4q22,) is found in intron 4 of the FAM13A gene and has no known biological function [43], [63]. The FAM13A protein, expressed in respiratory cells, is thought to be involved in signal transduction with possible tumor suppressor activity [63], [64]. The rs1422795 SNP (ADAM 19 on 5q33,) is a missense mutation (Ser284Gly) in the ADAM 19 gene. ADAM 19 is a transmembrane protein expressed in human lung implicated in cell-matrix interactions [65], pulmonary inflammation [66] and lung cancer [67]. The rs402710 SNP (CRR9 (TERT) on 5p15,) is an intronic SNP of unknown function in the CRR9 gene and associated with lung cancer in many studies [1], [17], [18], [34]. This SNP is 25 kb upstream from the TERT gene encoding, which encodes the catalytic subunit of telomerase, a reverse transcriptase that affects telomere shortening, which has been implicated in both aging and lung cancer [68]. The results of the current study suggest that the CRR9/TERT locus confers susceptibility to lung cancer in the absence of COPD. Such a finding is in accordance with those recently reported by Yang et al [34], who found after adjusting for the presence of COPD, only the rs 402710 SNP (Chr5p15 locus) was associated with lung cancer while the effects of the other GWA associated SNPs were lost. The rs2808630 SNP (CRP on 1q23,) is found in the 3′ flanking region of the CRP gene and has been associated with serum CRP levels (C allele with reduced CRP) [69]. Elevated CRP levels have been shown in prospective studies to be associated with greater decline in lung function [70] and elevated lung cancer risk after adjustment for smoking [71]. In the current study, where all cohorts were matched for smoking exposure, the CC genotype (low CRP level) was less frequent in both COPD and lung cancer cases although only achieved significance in the lung cancer only sub-phenotype. The rs2070600 SNP (AGER on 6p21,) is a missense mutation (Gly82Ser) of the AGER gene and shown to affect the inflammatory response in humans [72]. AGER protein expression has been shown to be increased in the lungs of smokers with COPD [73] whilst decreased in human lung cancer cell lines [74]. We conclude that the SNP associations described here with COPD and/or lung cancer can be explained by plausible, but as yet unproven, biological functions. We also conclude that through sub-phenotyping for COPD, possible clues as to the independent and overlapping pathogenic processes underlying COPD and lung cancer can be better examined.

The use of healthy smokers as controls in this study represents a novel though possibly controversial approach [31] to identifying the genetic basis of lung cancer. The authors contend that such an approach is classically used in pharmacogenetic studies where the disparate response to a standardised dose of drug provides a dynamic phenotype (high vs low metabolisers or responders vs nonresponders) from which to identify relevant genes [75]. In the setting of lung cancer, smoking is the drug and FEV_1_ the biomarker of responsiveness. The latter is based on the epidemiological studies showing that FEV_1_ is the most important risk factor for lung cancer among smokers [8], [9], [12], [8], [25,76] and has a bimodal distribution among chronic smokers [10]–[12]. The latter is very relevant as bimodal distribution supports a genetic basis as suggested by twin studies where heritability of FEV_1_ is estimated to be 40–77% compared to only 15–25% for lung cancer [6], [7]. From a genetic epidemiology perspective, a cohort of chronic smokers with the resistant or “non-responder” phenotype (normal or near normal FEV_1_), might provide an alternate control group to the non-random (and unscreened) smokers used in case-controls to date [17]–[19]. Controls recruited from hospital clinics or in the absence of spirometric screening (volunteers), report a COPD prevalence of 30% or more [33]). If the control group includes a high proportion of smokers with COPD, the effect of the COPD related genes on lung cancer susceptibility will be diluted or lost. This is also relevant as the proportion of COPD patients who eventually develop lung cancer may be as high as 25–30% [8], [77] and the frequencies of several disease-related SNPs are very similar between lung cancer and COPD groups (See Table 3, eg FAM13A, HHIP). This might explain why the lung cancer GWA studies to date failed to consistently identify the Chr4q31 (HHIP/GYPA) and Chr4q22 (FAM13A) loci as a protective loci [17]–[19], and the Chr 5q33 (ADAM19) locus as a possible susceptibility locus. It would also explain why matching for COPD in the lung cancer cases and controls might identify only the Chr5p21 (CRR9/TERT) locus which in the current study was associated with lung cancer in smokers with no underlying COPD [34]. The authors propose that FEV_1_ be routinely measured in genetic epidemiology studies of lung cancer to better understand the role of “COPD genes” in lung cancer [8], [12]. Subtyping for emphysema using computerised tomography or reduced diffusion capacity would further refine the subphenotyping for COPD [78].

It is possible that the specific associations reported in this study reflect in part, small sample size and chance findings. This represents an important limitation of the current study requiring replication in a larger study. It is also possible that the findings reflect true associations that have been better identified, despite small sample sizes, by more precise phenotyping of subjects. Minimising misclassification has been shown to improve the power of a study to identify true associations [36]. The authors suggest that some important associations may be either missed [18], [19] or miss-assigned [17]–[19] in studies where the COPD status of smoking controls is unknown, especially using hospital based controls where the prevalence of COPD has been found to be as high as 30% [33]. The latter would be analogous to searching for type 2 diabetes genes by comparing obese patients with type 2 diabetics thereby missing the genetic effects contributing to obesity. If previous case-control studies use control groups where the prevalence of COPD is 25–30%, then relevant genetic effect may be obscured. This is well illustrated in Table 3 where, for several SNPs (eg HHIP, GYPA, CRR9 (TERT), ADAM19 and CHRNA 3/5), the frequencies of “risk genotypes” between COPD and lung cancer cases are very similar. In addition, matching of other confounding variables, in particular smoking dose exposure, may also help to detect relevant genetic associations which might otherwise be diluted by using unexposed people (non-smokers [17]–[19]). Matching for smoking is particularly important in these studies of smoking related disease as the penetrance of SNP effects, reflected in the odds ratio, are likely to be related to the degree and/or duration of smoking exposure. The effect of certain SNPs have been shown to be greater when investigated only in those with greater smoking exposure [21], [29]. This is the case in α1-anti-trypsin deficiency where people homozygote for the Z allele (low α1-antitrypsin level) are at risk of emphysema when they smoke, but much less so when they are non-smokers [79]. Lastly, there remains the possibility that the SNP associations reported here result from gender, age or height differences between the group comparisons. Although our sample sizes are modest, we think this is unlikely as the groups are comparable with respect to these variables and we specifically examined this possibility and did not find any SNP effects confounded by these variables.

The authors have previously reported a lung cancer susceptibility model whereby genotype data is combined with non-genetic data [27], [32]. This model is based on the results of a multivariate analysis that include the genotypes, scored according to whether they conferred a small protective (-1) or susceptibility (+1) effect [27], [32]. The clinical variables, identified as independent predictors of lung cancer following multivariate analysis were, age over 60 years, a family history of lung cancer and previous diagnosis of COPD. In stepwise regression, family history of lung cancer is independently associated with lung cancer risk after inclusion of the SNP genotype data [80] and likely reflects rare family-specific genetic effects not accounted for by the genotypes tested here. An example of such a genetic effect is represented by the RGS17 gene on Chr 6q24 implicated in familial lung cancer but not investigated here [81]. Similarly, the prior diagnosis of COPD is independently associated with lung cancer risk and likely reflects the contribution of genetic susceptibility to COPD not otherwise accounted for by the SNPs in the panel. The SNP data provides an important and significant contribution to the overall score as “risk genotypes” are a risk variable present from birth, and unlike family history and diagnosis of COPD, not dependent on age or natural history of disease. This is very relevant to prevention as high risk SNP genotypes can be identified early in a person's smoking history, before irreversible malignant transformation has occurred. Although lung function data itself is also an important variable in defining the risk of lung cancer, it is usually not available for the majority of smokers where it is often not done until exertional breathlessness is severe and when over 50% of lung function is irreversibly lost [12]. For each subject in the control smoker and lung cancer cohorts, a lung cancer susceptibility score was derived according to these variables and their distributions compared [27], [32]. The distribution showed a bimodal separation suggesting utility as a screening test of risk [27], [32], [82]. Using the same approach in the current study, with the susceptibility and protective genotypes derived from the GWA SNPs (9 SNP panel, Table 4), the lung cancer susceptibility score was also bimodal and showed a limited utility in an ROC analysis (AUC = 0.69) (Figures 2 and 3). This utility was increased when the 10 most informative SNPs from the previous study were added (N = 19 SNP model, AUC = 0.72, data not shown). This suggests that as new genetic variants are identified and added to the risk model, a greater utility based on ROC analysis might be achieved [31], [80]. This study provides further evidence that lung cancer results from the combined effects of several genetic variants [83] with low penetrance [84] from genes implicated in both COPD and lung cancer [26]–[28]. This study also highlights the limitations of the lung cancer GWA studies reported to date [85] and the need to consider sub-phenotyping using spirometry-defined COPD to better understand the relative effects of genetic variants on lung cancer susceptibility [26], [28]. In conclusion, this study provides additional evidence that genes involved in the risk of COPD may also be relevant to the risk of lung cancer and that spirometry be routinely used to identify COPD, an important sub-phenotype of lung cancer. This study also supports the potential of combining genotype data [27], [32] in an algorithmic fashion to identify smokers at greatest risk of lung cancer.

## References

[1] Broderick P, Wang Y, Vijayakarishnan J, Matakidou A, Spitz MR (2009). Deciphering the impact of common genetic variation on lung cancer risk: A genome-wide association study.. Cancer Res;.

[2] Molfino NA (2004). Genetics of COPD.. Chest;.

[3] Mattson ME, Pollack ES, Cullen JW (1987). What are the odds that smoking will kill you?. Am J Pub Health.

[4] Kohansal R, Martinez-Camblor P, Agusti A, Buist AS, Mannino DM (2009). The natural history of chronic airflow obstruction revisited: An analysis of the Framingham Offspring Cohort.. Am J Respir Crit Care Med.

[5] Løkke A, Lange P, Scharling H, Fabricius P, Vestbo J (2006). Developing COPD: A 25 year follow up study of the general population.. Thorax.

[6] Lichtenstein P, Holm NV, Verkasalo PK, Iliadou A, Kaprio J (2000). Environmental and heritable factors in the causation of cancer: Analyses of cohorts of twins from Sweden, Denmark and Finland.. N Eng J Med.

[7] Redline S, Tishler PV, Rosner B, Lewitter FI, Vandenburgh M (1989). Genotypic and phenotypic similarities in pulmonary function among family members of adult monozygotic and dizygotic twins.. Am J Epidemiol.

[8] Young RP, Hopkins RJ, Christmas T, Black PN, Metcalf P (2009). COPD prevalence is increased in lung cancer independence of age, gender and smoking history.. Euro Respir J.

[9] Mannino DM, Aguayo SM, Petty TL, Redd SC (2003). Low lung function and incident lung cancer in the United States: Data from the first NHANES follow-up.. Arch Int Med.

[10] Burrows B, Knudson RJ, Cline MG, Lebowitz MD (1977). Qualitative relationships between cigarette smoking and ventilatory function.. Am Rev Respir Dis.

[11] Dockery DW, Speizer FE, Ferris BG, Ware JH, Louis TA (1988). Cumulative and reversible effects of lifetime smoking on simple tests of lung function in adults.. Am Rev Respir Dis.

[12] Young RP, Hopkins RJ, Eaton TE (2007). Forced expiratory volume in one second: Not just a lung function test but a marker of premature death from all causes.. Eur Respir J.

[13] Wilson DO, Weissfeld JL, Balkan A, Schragin JG, Fuhrman CR (2008). Association of radiographic emphysema and airflow obstruction with lung cancer.. Am J Respir Crit Care Med.

[14] de Torres J, Bastarrika G, Wisnivesky JP, Alcaide AB, Campo A (2007). Assessing the relationship between lung cancer risk and emphysema detected on low dose CT of the chest.. Chest.

[15] Mortensen EM, Copeland LA, Pugh MJ, Fine MJ, Nakashima B (2010). Diagnosis of pulmonary malignancy after hospitalisation for pneumonia.. Am J Med.

[16] Wilk JB, Walter RE, Laramie JM, Gottlieb DJ, O'Connor GT (2007). Framingham heart study genome-wide association: Results for pulmonary function measures.. BMC Med Genet.

[17] Amos CI, Wu X, Broderick P, Gorlov IP, Gu J (2008). Genome-wide association scan of tag SNPs identifies a susceptibility locus for lung cancer at 15q25.1.. Nature Genetics.

[18] Hung RJ, McKay JD, Gaborieau V, Boffetta P, Hashibe M (2008). A susceptibility locus for lung cancer maps to nicotinic acetylcholine receptor subunit genes on 15q25.. Nature.

[19] Thorgeirsson TE, Geller F, Sulem P, Rafnar T, Wiste A (2008). A variant associated with nicotine dependence, lung cancer and peripheral arterial disease.. Nature.

[20] Pillai SG, Ge D, Zhu G, Kong X, Shianna KV (2009). A genome-wide association study in chronic obstructive pulmonary disease (COPD): Identification of two major susceptibility loci.. PLoS Genetics.

[21] Wilk JB, Chen T, Gottlieb DJ, Walter RE, Nagle MW (2009). A genome-wide association study of pulmonary function measures in the Framingham Heart Study.. PLoS Genetics.

[22] Repapi E, Sayers I, Wain LV, Burton PR, Johnson T (2009). Genome-wide association study identifies five loci associated with lung function.. Nature Genetics.

[23] Hancock DB, Eijgelsheim M, Wilk JB, Gharib SA, Loehr LR (2009). Meta-analyses of genome-wide association studies identify multiple loci associated with pulmonary function.. Nature Genetics.

[24] Petty TL (2005). Are COPD and lung cancer two manifestations of the same disease?. Chest.

[25] Schwartz AG, Ruckdeschel JC (2006). Familial lung cancer: Genetic susceptibility and relationship to chronic obstructive pulmonary disease.. Am J Respir Crit Care Med.

[26] Young RP, Hopkins RJ, Hay BA, Epton MJ, Black PN (2008). Lung cancer gene associated with COPD: Triple whammy or possible confounding effect?. Eur Respir J.

[27] Young RP, Hopkins RJ, Hay BA, Epton MJ, Mills GD (2009). Lung cancer susceptibility model based on age, family history and genetic variants.. Plos One.

[28] Young RP, Whittington CF, Hopkins RJ, Hay BA, Epton MJ (2010). Chromosome 4q31 locus in COPD also associated with lung cancer.. Eur Respir J.

[29] Punturieri A, Szabo E, Croxton TL, Shapiro SD, Dubinett SM (2009). Lung cancer and chronic obstructive pulmonary disease: Needs and opportunities for integrated research.. JNCI.

[30] Frayling TM (2007). Genome-wide association studies provide new insights into type 2 diabetes aetiology.. Nat Reviews Genet.

[31] Janssens ACJW, van Duijn CM (2009). Genome-based prediction of common disease: methodological considerations for future research.. Genome Med.

[32] Young RP, Hopkins RJ, Hay BA, Epton MJ, Mills GD (2009). A gene based risk score for lung cancer susceptibility in smokers and ex-smokers.. Postgrad Med J.

[33] Stav D, Raz M (2007). Prevalence of chronic obstructive pulmonary disease among smokers aged 45 and up in Israel.. Isr Med Assoc J.

[34] Yang P, Yafei L, Jiang R, Cunningham JM, Zhang F (2010). A rigorous and comprehensive validation: Common genetic variations and lung cancer.. Cancer Epidemiol Bio Prev.

[35] Lambrechts D, Buysschaert I, Zanen P, Coolen J, Lays N (2010). The 15q24/25 susceptibility variant for lung cancer and COPD is associated with emphysema.. Am J Resipr Crit Care Med.

[36] Moskvina V, Holmans P, Schmidt KM, Craddock N (2005). Design of case-controls studies with unscreened controls.. Ann Hum Genet.

[37] Zheng SL, Sun J, Wiklund F, Smith S, Stattin P (2008). Cumulative association of five genetic variants with prostate cancer.. NEJM.

[38] Easton DF, Peto J, Babiker AGAG (1991). Floating absolute risk: an alternative to relative risk in survival and case-control analysis avoiding an arbitrary reference group.. Statistics in Medicine.

[39] Plummer M (2004). Improved estimates of floating absolute risk.. Statistics in Medicine.

[40] Pritchard J, Stephens M, Donnelly P (2000). Inference of population structure from multilocus genotype data.. Genetics.

[41] Thankkinstian A, Thompson JR, Minelli C, Attia J (2009). Choosing between per-genotype, per-allele, and trend approaches for initial detection of gene-disease association.. J App Stat.

[42] Yang P, Allen MS, Aubry MC, Wampfler JA, Marks RS (2005). Clinical Features of 5,628 Primary Lung Cancer Patients: Experience at Mayo Clinic from 1997 to 2003.. Chest.

[43] Jin G, Xu L, Shu Y, Tian T, Liang J (2009). Common genetic variants on 5p15.33 contribute to risk of lung adenocarcinoma in a Chinese population.. Carcinogenesis.

[44] Young RP, Hopkins R, Eaton TE (2009). Pharmacological actions of statins: potential utility in COPD.. Eur Respir Rev.

[45] Brown V, Elborn JS, Bradley J, Ennis M (2009). Dysregulated apoptosis and NFĸB expression in COPD subjects.. Resp Research.

[46] Sohal SS, Reid D, Soltani A, Ward C, Weston S (2009). Smoking has potential to initiate basement membrane disruption and epithelial mesenchymal transition in COPD..

[47] Dasari V, Gallup M, Lemjabbar H, Maltseva I, McNamara N (2006). Epithelial-mesenchymal transition in lung cancer: Is tobacco the “smoking gun”?. Am J Respir Cell Mol Biol.

[48] Spitz MR, Wei Q, Dong Q, Amos CI, Wu X (2003). Genetic susceptibility to lung cancer: The role of DNA damage and repair.. Cancer Epidemiol Biomarkers Prev.

[49] Shintani Y, Maeda M, Chaika N, Johnson KR, Wheelock MJ (2008). collagen 1 promotes epithelial-to-mesenchymal transition in lung cancer cells via transforming growth factor-β signalling.. Am J Respir Cell Mol Biol.

[50] Zondag GCM, Evers EE, ten Klooster JP, Janssen L, van der Kammen RA (2000). Oncogenic Ras downregulates rac activity, which leads to increased Rho activity and epithelial-mesenchymal transition.. J Cell Biol.

[51] Lee G, Walser TC, Dubinett SM (2009). Chronic inflammation, chronic obstructive pulmonary disease and lung cancer.. Curr Opin Pulm Med.

[52] Weiss KM, Clark AG (2002). Linkage disequilibrium and the mapping of complex human traits.. Trends Genet.

[53] Dickson SP, Wang K, Krantz I, Hakonarson H, Goldstein DB (2010). Rare variants create synthetic genome-wide associations.. PLoS Biol.

[54] Gwilt CR, Donnelly LE, Rogers DF (2007). The non-neuronal cholinergic system in the airways: An unappreciated regulatory role in pulmonary inflammation?. Pharmacol Therapeut.

[55] Schuller HM (2007). Nitrosamines as nicotinic receptor ligands.. Life Sci.

[56] Bierut LJ, Stitzel JA, Wang JC, Hinrichs AL, Grucza RA (2008). Variants in nicotinic receptors and risk for nicotine dependence.. Am J Psychiatry.

[57] Rudd MF, Webb EL, Matakidou A, Sellick GS, Williams RD (2006). Variants in the GH-IGF axis confer susceptibility to lung cancer.. Genome Res..

[58] Sasaki T, Gan EC, Wakeham A, Kornbluth S, Mak TW (2007). HLA-B-associated transcript 3 (BAT3)/Scythe is essential for p300-mediated acetylation of p53.. Genes and Development.

[59] Shi W, Chen F, Cardoso WV (2009). Mechanisms of lung development: Contribution to adult lung disease and relevance to chronic obstructive pulmonary disease.. Proc Am Thorac Soc.

[60] Watkins DN, Berman DM, Burkholder SG, Wang B, Beachy PA (2003). Hedgehog signalling within airway epithelial progenitors and in small-cell lung cancer.. Nature.

[61] Shintani Y, Maeda M, Chaika N, Johnson KR, Wheelock MJ (2008). Collagen I promotes epithelial-to-Mesenchymal Transition in lung cancer cells via transforming growth factor- signalling.. Am J Respir Cell Mol Biol.

[62] Minetti M, Leto TL, Malorni W (2008). Radical generation and alterations of erythrocyte integrity as bio-indicators of diagnostic or prognostic value in COPD?. Antioxidants & Redox Signaling.

[63] Cho MH, Boutaoui N, Klanderman BJ, Sylvia JS, Ziniti JP (2010). Variants in FAM13A are associated with chronic obstructive pulmonary disease.. Nature Genetics.

[64] Young RP, Hopkins RJ, Hay BA, Whittington CF, Epton MJ (2011). FAM13A locus in COPD independently associated with lung cancer – evidence of a molecular genetic link between COPD and lung cancer.. App Clin Genet.

[65] Arribas J, Bech-Serra JJ, Santiago-Josefat B (2006). ADAMs, cell migration and cancer.. Cancer Metastasis Rev.

[66] Dijkstra A, Postma DS, Noordhoek JA, Ladewijk ME, Kauffman HF (2009). Expression of ADAMs (“a disintegrin and metalloprotease”) in the human lung.. Virchows Arch.

[67] Qi B, Newcomer RG, Sang QXA (2009). ADAM19/adamalysin 19 structure, function, and role as a putative target in tumors and inflammatory diseases.. Current Pharmaceutical Design.

[68] Rafnar T, Sulem P, Stacey SN, Geller F, Gudmundsson S (2009). Sequence variants at the TERT-CLPTM1L locus associate with many cancer types.. Nature Genetics.

[69] Crawford DC, Sanders CL, Qin X, Smith JD, Shephard C (2006). Genetic variation is associated with C-reactive protein levels in the third national health and nutrition examination survey.. Circulation.

[70] Rasmussen F, Mikkelsen D, Hancox RJ, Lambrechtsen J, Nybo M (2009). High-sensitive C-reactive protein is associated with reduced lung function in young adults.. Euro Respir J.

[71] Allin KH, Bojesen SE, Nordestgaard BG (2009). Baseline C-reactive protein is associated with incident cancer and survival in patients with cancer.. J Clin Oncol.

[72] Hofmann MA, Dury S, Hudson BI, Gleason MR, Qu W (2002). RAGE and arthritis: the G82S polymorphism amplifies the inflammatory response.. Genes Immun.

[73] Ferhani N, Letuve S, Kozhich A, Thibaudeau D, Grandsaigne M (2010). Expression of High-Mobility Group Box 1 and of Receptor for Advanced Glycation End products in Chronic Obstructive Pulmonary Disease.. Am J Resipr Crit Care Med.

[74] Bartling B, Hofmann HS, Weigle B, Silber R-E, Simm A (2005). Down-regulation of the receptor for advanced glycation end-products (RAGE) supports non-small cell lung carcinoma.. Carcinogenesis.

[75] Weinshilboum R (2003). Inheritance and drug response.. N Eng J Med.

[76] Tockman MS, Anthonisen NR, Wright EC, Donithan MG (1987). Airways obstruction and the risk for lung cancer.. Annal Int Med.

[77] Anthonisen NR, Connett JE, Enright PL, Manfreda J (2002). Hospitalizations and mortality in the Lung Health Study.. Am J Resipr Crit Care Med.

[78] Weatherall M, Travers J, Shirtcliffe PM, Marsh SE, Williams MV (2009). Distinct clinical phenotypes of airways disease defined by cluster analysis.. Eur Respir J.

[79] Piitulainen E, Eriksson S (1999). Decline in FEV1 related to smoking status in individuals with severe alpha1-antitrypsin deficiency (PiZZ).. Eur Respir J.

[80] Wright CF, Kroese M (2010). Evaluation of genetic tests for susceptibility to complex diseases: why, when and how?. Hum Genet.

[81] You M, Wang D, Liu P, Vikis H, James M (2009). Fine mapping of chromosome 6q23-25 region in familial lung cancer families reveals RGS17 as a likely candidate gene.. Clin Cancer Res.

[82] Wald NJ, Hackshaw AK (1999). When can a risk factor be used as a worthwhile screening test?. Brit Med J.

[83] Xu H, Spitz MR, Amos CI, Shete S (2005). Complex segregation analysis reveals a multigene model for lung cancer.. Hum Genet.

[84] Shields P, Harris C (2000). Cancer risk and low-penetrance susceptibility genes in gene-environment interactions.. J Clin Oncol.

[85] Pearson TA, Manolio TA (2008). How to interpret a genome-wide association study.. JAMA.

